# Social and cultural complexities of anti-malarial drug circulation: an ethnographic investigation in three rural remote communes of Cambodia

**DOI:** 10.1186/s12936-017-2082-7

**Published:** 2017-10-25

**Authors:** Phasy Res

**Affiliations:** 1 Durham University, Durham, UK; 2 Institute de Recherche pour le Développement (IRD), P.O.Box 591, Phnom Penh, Cambodia

**Keywords:** Anti-malarial drug resistance, Artemisinin Combination Therapy, Choice of treatments, Public and private sector, Cambodia

## Abstract

**Background:**

Anti-malarial medicine has a central role in malaria case management in Cambodia. It is, therefore, essential to study how anti-malarial drugs are distributed and consumed. This study aims to understand the socio-cultural complexity of anti-malarial drugs provision and usage practices.

**Methods:**

Semi-structured interviews and observation were conducted in Cambodia at the communal, provincial, and national levels from January 2014 to January 2015. Health ministers, non-governmental officers, anti-malarial medicines distributors, village malaria volunteers and malaria patients were interviewed.

**Findings:**

The findings show that artemisinin-based combination therapy (ACT) flows into unregulated outlets, and was sold without any diagnostic tests. Affordable Medicines Facility for malaria scheme (AMFm) cannot drive ineffective anti-malarial medicines out of the market because ACT is still more expensive due to price absortion by private and public providers. Malaria patients might not consume ACT because of patients’ notions of ‘*Korp*’, and pharmaceutical and parasitic familiarity. The findings reflect that neither public nor private institutions have the capacity and resources to control the flow of ACT from going into the unlicensed sector. They do not have the ability to ensure that ACT is consumed after a positive rapid diagnostic test.

**Conclusions:**

With a weak regulation system and ailing public health infrastructure, pharmaceutical-neoliberal mechanism like AMFm is not an effective means to eradicate any forms of malaria. Therefore, horizontal programmes, such as public health infrastructure improvement, and population participation must be implemented. Ethnical responsibilities of medical practitioners must be enforced and be included into the national curriculum. The awareness of drug resistance must be implemented at all levels.

## Background

In many developing countries, Malaria has been a persistent and critical public health problem. Many Asian countries, including Cambodia, are signatories to the UN’s Millennium Development Goals, eliminating all forms of malaria infection by 2025 [1]. However, this goal is undermined by the persistent nature of anti-malarial drug resistance of the *Plasmodium falciparum* parasite. The Global Fund to Fight AIDS, Tuberculosis and Malaria (GFATM) and World Health Organization (WHO) have implemented many mechanisms to combat the recurrence of anti-malarial drug resistance. For example, the WHO in collaboration with Cambodian and Thai Ministries of Health started a containment programme in 2009, launching a containment project aimed at preventing the development of artemisinin drug resistance in malaria-infested areas, along the Thai-Cambodian border [2]. Later on, GFATM mobilized its financial resources from the Roll Back Malaria Partnership, the World Bank, the UK Department for International Development, Joint United Nations Programme on HIV/AIDS (UNITAID), and the Bill & Melinda Gates Foundation [3] to support its initiative called Affordable Medicine Facility Malaria (AMFm). AMFm subsidizes ACT to malaria endemic countries, in order to lower the price of ACT so that it will drive out ineffective anti-malarial drugs for *P. falciparum*, (deadly parasite), such as chloroquine, sulfadoxine-pyrimethamine or artemisinin monotherapy [4].

Cambodia was of the pilot countries of the initiative. ACT is strictly monitored by three public and private bodies, National Centre for Parasitology, Entomology and Malaria Control (CNM), Population Service Khmer (PSK) or formerly known as Population Service International (PSI), and University Research Cooperation (URC). According to the national malaria guideline, the three bodies only distribute ACT to the outlets that signed memorandum of understanding (MoU) with them so that they can strictly monitor the ACT provision [5].

The WHO containment project and GFATM’s AMFm initiative place anti-malarial medicine as one of the core strategies to fight malaria. This shapes malaria guideline policies in endemic countries. For the last three decades, malaria strategies plans in Cambodia have been situated around vectors and parasites control. In practice, this meant distributing insecticide-treated bed nets, and shifting to the most updated anti-malaria drugs. Before 2000, the national guideline for uncomplicated *P. falciparum* was a single dose of mefloquine, and a combination of quinine and tetracycline for the treatment of complicated malaria [6]. However, tests in 1994 and 1995 showed that throughout the country, malaria parasites were sensitive to mefloquine, quinine, chloroquine and tetracycline [7]. In 2005, the *P. falciparum* parasitic resistance to all form of mono-therapies became a threat in western, north-western and north-eastern Cambodia [8–10]. By 2006, the WHO strongly recommended all malaria endemic countries to switch from mono-therapies, such as chloroquine, amodiaquine, and sulfadoxine-pyrimethamine for the treatment of *falciparum* malaria, to ACT [11]. At the time of this research the co-formulation of ACT is dihydroartemisinin and piperaquine (DHA-PPQ) [2], which is also currently threatened by parasitic resistance. The above facts illustrate how malaria eradication mechanisms avoid any political engagements such as improving public health care infrastructure or strengthening the regulation of pharmaceuticals.

In late April 2017, the WHO reported a sign of *P. falciparum* parasitic resistance to artemisinin and its partner drugs [12]. Artemisinin is the most effective substance in treating malaria, so this poses a major threat to the global public health’s effort to eliminate malaria. The threat of resistance to artemisinin is still under the WHO’s close surveillance. If there is enough evidence to confirm artemisinin resistance, the WHO proposes to discuss alternatives to non-ACT treatment options [ibid]. The understanding of resistance is predominantly situated around parasite-anti-malarial medicines interaction, which places the medicine as one of the core strategies to combat malaria. Bieh [13] called such a solution a pharmaceutically-centered model of public health or a ‘magic bullet approach’ to health care.

In Cambodia, there is some prior research on parasitic-drug interaction. For example, a cohort study in Pursat, Preah Vihear, and Ratanakiri found that dihydroartemisinin–piperaquine (ACT) failures are caused by artemisinin and piperaquine resistance [14]. They propose artesunate plus mefloquine as an alternative ACT formula to treat uncomplicated *P. falciparum* in the area of dihydroartemisinin–piperaquine failure [ibid]. However, little research explores the interaction between patient-medicines’ and patients-health providers’ relations, and public–private practices of health providers. Therefore, this paper seeks to fill this gap by exploring the social and cultural complexities that shape anti-malarial drug provision and usage practices. The findings show that ACT is consumed without RDT, and chloroquine is still preferable to ACT. As this paper will argue, malaria usage and practices are also shaped by available health care infrastructure, ethical medical responsibilities, and people’s understanding of the body and disease. The remainder of the article illustrates these arguments in greater details.

## Methods

### Locations of research

The study was conducted in multi-sited fieldworks. The research was conducted with both urban and rural area, with the goals of understanding the flow of anti-malaria drugs from the most concentrated pharmaceuticals (Phnom Penh) to remote rural areas like Russey Chrum. The Olympic market area was selected as one of the locations to be studied because it was a pharmaceutical hub. This area is located in the centre of Phnom Penh, the capital city of Cambodia. Many pharmaceutical factory representatives, pharmaceutical companies, wholesalers and retailers are clustered together in that area. Based on the ethnographic investigation carried out in this research, there were approximately 93 pharmaceutical outlets and four pharmaceutical companies. At the local level, three communes were selected as target locations. They were, Steung Keo (Kampot province), Russey Chrum (Koh Kong province), and Pu Jrey (Mondulkiri province). They are chosen because of the prevalence of malaria, the high migration density, and the close interaction between the public and private sectors.

Steng Keo is a commune in Teuk Chu district, which is located at the foot of a mountain approximately 25 km from Kampot city, where the forest is very sparse. Incidence of malaria is relatively low, but it remains a serious issue because of high population mobility. The majority of the people depend on rice farming and collecting forest products. The out-migration of seasonal migrants is very high; people from Steung Keo tend to go to the forest in Koh Kong to collect forest products. Due to this migration, Steung Keo remains a malaria endemic area.

Russey Chrum is a commune in Thmor Bang district, located deep in the forest, about 70 km from Koh Kong city. Here the forest is still very dense despite extensive logging activities. Malaria infection rate is higher than in Steung Keo. Most people get infected because they regularly go to the forest nearby to collect timber and other forest products. The majority of people depend on subsistence farming and trading forest products. Russey Chrum used to be inhabited by Chong indigenous people before being used as a military base to fight against the Khmer Rouge. When the military base was abandoned, many ex-combatants from both sides of the conflict chose to stay there. Later on, more and more people migrated to Thmor Bang district so that they could get a piece of land for farming, usually with the support of relatives and neighbours. Seasonal migrants also started to come to Thmor Bang to collect forest products. Most of the permanent and seasonal migrants are from Kampot, Sihanouk Ville, Kompong Cham, Battambang, Kompong Speu and Siem Reap province.

Pu Jrey is a commune of Peachda district located in Mondulkiri province. It is located in a hilly environment and it used to be surrounded by dense forests, which have recently been replaced by rubber plantations. Hence, the forests have become sparse over time. Similar to Russey Chrum, people in Pu Jrey mostly depend on commercial plantations, subsistence crop farming and forest products for their livelihood. Malaria is prevalent in this area. The commune used to be inhabited by only the indigenous Bunong people. After the demise of the Khmer Rouge, the Cham ethnic minority started to come in and acquire huge pieces of land for rubber and cassava plantations. This was followed by the arrival of more and more Khmer people from nearby provinces, such as Prey Veng, Kompong Cham and Kratie. Industrial and commercial plantations have also attracted many seasonal migrant workers. Due to internal migration, Steung Keo, Russey Chrum and Pu Jrey are interconnected.

### Data collection

Ethnographic investigation, semi-structured interviews, informal conversations and group discussions were carried out. Data was collected between January 2014 and January 2015. Primary data was collected from 73 in-depth interviews with high-ranking health officials, NGO officials, drug inspectors, pharmacists, local health care providers, Villager Malaria Workers (VMWs), malaria patients and their family members. The average duration of an interview was 50 min, but interviews ranged from 40 min to 2 h. Interviews were mostly conducted with men, as the majority of NGO workers on the topics were men, and men often moved back and forth between the forest and the village, as a result they were infected with malaria. Moreover, ethnographic observations were conducted at each health centre for 3 h per day for five consecutive days (one centre per province). The observation was also conducted at six private outlets for the same amount of time. The aim was to understand the general malaria treatment practices, as well as the flow of the medicines from the health centre to the patients. In that context, the heterogeneity of the three health centres’ practices was observed. Moreover, staff from these health centres was also observed in their private practices.

### Data analysis

At first, a tape recorder was used to record villagers’ and malaria patients’ group discussions, as well as interviews with medical providers and health centre staff. The recording was then transcribed and translated from Khmer into English. However, when the discussion touched upon sensitive issues, only hand-written notes were taken. When the conversation became even more confidential and sensitive, the author refrained from taking notes in front of the participant. This flexible technique of capturing field information was deliberately deployed so as to make the participants feel as comfortable as possible, without compromising the accuracy and validity of the data collected. The data was analysed manually using qualitative content analysis, coding according to themes. Several statements expressed by the study participants were quoted, some representing a common view, experience, or practices.

## Results

### The flow of ACT to the unregistered and unregulated sector

Observation showed that unregulated and unregistered outlets can buy ACT medicines from registered outlets, a point also noted by Ovesen and Trankell [15]. For instance, in a remote rural area of Koh Kong, a private unlicensed medical provider told the author that because PSK refused to distribute ACT to her, she bought them from a licensed wholesale pharmacy in Koh Kong city instead. ACT can also flow directly from the Olympic Market (Phnom Penh) to provincial level retailers. A private provider in Mondulkiri said that she ordered ACT from Olympic market (12 packs for US$14). According to a CNM officer, ACT is not allowed to be available at the Olympic pharmaceutical hub. It was acknowledged by the GFATM that between 2009 and 2011, nearly US$ 2.3 million worth of donated anti-malaria medicines were stolen from government-run warehouses in Cambodia, Kenya, Nigeria, Sierra Leone, Swaziland, Tanzania and Togo [16]. Moreover, according to the interview with the CNM officer, in 2012 the Cambodian anti-economic crime police found more than 10,000 packs of public anti-malaria medicines (A + M) in a private warehouse in Phnom Penh. Once ACT is available in Olympic pharmaceutical hub, it will be sold and consumed without any monitoring processes, and the benefit of lower cost of ACT is being diverted from the poor.

### The sale of ACT without diagnostic tests

ACT is sold without any diagnostic tests, which can create drug pressure. This increases the chance of ACT resistance. The author found that 12 packs of ACT medicine were sold at the cost of US$25 in wholesale pharmacies in Phnom Penh to anyone who asks for it without inquiring about the diagnostic test. At the time of this research in early 2014, there was an influx of expired anti-malaria medicines available in Cambodia. The private medical providers in Kompot, Koh Kong, and Mondulkiri did not hesitate to sell these medicines directly without conducting any blood tests. This practice can be seen as an opportunist behaviour of medical providers who want to reap some profits by selling expired or near expired anti-malaria medicines as soon as possible. Moreover, according to author’s observation at private outlets, a provider sold ACT without any diagnostic tests. Prescribing ACT after a positive RDT is crucial as Manyando et al. [17] claims it as a way to achieve malaria elimination.

### The price absorption of ACT by public and private providers

AMFm promises to lower the price of ACT to drive out the ineffective anti-malarial medicines for *P. falciparum* like chloroquine out of the market, so that malaria patients can get high quality and cheap anti-malarial medicines. However, the AMFm scheme does not address the problem of retailer price absorption. Many researchers, including Harford have voiced this problem. He has warned that if the system is ‘leaky’, the benefits of the price subsidy would go to the retailers instead of the consumers [18]. The data showed that private outlets bought ACT at subsidized prices and sold them to consumers at a mark-up of 150%; which makes the price of ACT higher than the chloroquine, which cost US$ 0.20 [4]. Similarly, the finding of this paper showed that retailers charged US$ 2.5 US$, US$ 7, and up to US$ 10 for the full course of ACT while chloroquine cocktails were US$ 0.25 per intake. Normally patients consume three intakes when they are having shivering fever prior to any diagnostic tests.

Moreover, malaria patients are supposed to get ACT medicine at any health centre or health post cheaply (US$ 0.25), but they cannot because of public–private practices. Public health officers take advantage of the public position, to sell the publicly subsidized ACT over the private counter. A significant number of public health workers were actually operating in both sectors. This undoubtedly facilitated the flow of anti-malarial medicines from the public sector to the private sector, particularly when these health workers who have access to these medicines in the public sector, sell them over their private counters. These public health workers who also work as private vendors do not consider their practice unethical because this behaviour has been normalized over time. They justified their practice by pointing to their low salary as public health workers. For example, health centre staff from two different research locations told the author that ‘if you want the cow to work, you need to provide enough grass’, meaning that the salary has to be higher if health centre staff are expected to perform their duties fully, and to follow all ethical principles (e.g. not taking public medicines to sell at private counters). During an observation at one of the health centres, a health centre staff said he used to take anti-malarial medicines from the health centre to cure patients who visited his private service. He charged his patients US $3 for a full course of treatment and paid US $0.25 to the health centre, which was the actual cost of the anti-malarial medicine. He insisted that he did not do anything wrong since he had paid the cost of the medicine to the health centre.

### Tasteless saliva ‘Tek Mort Sarp’

In response to the public–private practices, villagers often complain, but they do not dare to speak up. During one of the regular group discussions in the course of this research, villagers were complaining about the absence of health workers and the neglect by staff from the Health Centres. However, the villagers were too fearful to raise the problems during the meeting run by the NGO, CARE Cambodia. The aim of CARE Cambodia was to empower villagers to speak up, but one of them later confided that she dared not to do so at the communal meetings for fear of recriminations from the medical practitioners afterwards. She feared that they might retaliate by refusing to treat her or her family members in their private or public practices.

Her fears are well founded. In a remote rural area, such as Russey Chrum, the Health Centre staff is typically the main medical practitioner in both the public, and private sectors. Other villagers shared similar opinions, noting that even if they did complain, it did not mean that the Health Centre staff would listen to them. Villagers described this as their “Tek Mort Sarp”, which literally means tasteless saliva, in contrast with salty saliva. If a person said his or her saliva is tasteless in Khmer language, it implies that whatever s/he said is meaningless or s/he is powerless to change the situation.

These public–private practices shape patients’ expectations of the malaria treatment. According to in-depth interviews and group discussions conducted with villagers who have experienced malaria infections, they preferred perfusion and vitamins to go along with malaria treatment. However, at the Health Centres, they were only given a package of ACT and paracetamol. Thus, many patients were dissatisfied with such meager malaria treatment. In Cambodia, the perception that perfusion works better and treats faster is a widespread belief since the early introduction of biomedicine in colonial times [15]. Cambodians believe that perfusion reduces the side-effects of anti-malaria medicines, and vitamins help them recover faster.

Many pharmaceutical anthropologists are trying to understand the attitudes of medical practitioners and patients behind this practice. Some researchers regard such practice as an opportunistic behaviour of doctors and pharmacists, who take advantage of consumers’ ignorance about appropriate treatment [19]. Other claims it could be that “it is easier to satisfy the patients with drugs than with words” [20]. Thus ironically, many malaria patients are reluctant to seek a low-cost malaria treatment at the health centres, instead they seek treatment from the private sector which might not necessary be monitored by the authorities.

Such expectations are enforced by the dual practices of medical providers. Some public health staff enforced this expectation by making the treatment at the private sector superior to the public sector’s. Some public–private medical practitioners perceive perfusion as unnecessary for malaria treatment, but would administer it in their private practice anyway. A staff member at the Health Centre who was interviewed by the author explained that perfusion is just like water, it is not needed for all patients or in all treatments. Thus, the Health Centres would consider administering perfusion or vitamins to malaria patients as unnecessary if the malaria symptoms are not severe. However, according to an interview with a malaria patient who got treatment from this staff member in his private practice, the patient confirmed that perfusion was used for his treatment. By doing so, it gave the patients the impression that private services are somehow superior or more effective than public services. As Nichter [21] claims, “when the government doctors carry out private practice after hours in which they provide different types of medications, they either intentionally or inadvertently contribute to the impression that the standard of government care is inferior”. This therefore, discouraged people to seek malaria treatment from the public sector, even though high quality and cheap medicines like ACT are available there.

### The body conception of *Korp*

In some cases, the patient’s choice of treatment is determined not by their expectation or the efficacy of the medicine, but by their conception of the body in relation to modern health services. Many Cambodians express the effectiveness of medicines through the concepts of *Korp* (fit/match) or *Ort Korp* (do not fit/match). This belief is strongly associated with individual practitioners or institutions like health centres. It basically refers to the compatibility between the patients and the health practitioner or type of service in question. It is an old traditional concept that is also found in the Philippines. Hardon who conducted a study in the Philippines, in which the informants had explained to him that the medicines prescribed should be compatible to the person using it, which in the local language, is referred to as *Hiyang* (as cited in [20]).

In the Cambodian case, it is slightly different. The concept of *Korp* is not dependent on the type of medicines but on the person(s) administering it, so patients or their family members would consider that there was *Korp* between them and a particular medical practitioner if he or she repeatedly cured them. In this way, the patients believe that their children or they themselves “*Korp”* with a particular medical provider; who can be either a licensed or unlicensed provider. This concept of *Korp* builds a strong relationship between the medical practitioners and the patients since the latter would regularly seek the former’s help. If a villager said his/her child *Korp* with this or that practitioner, it means that only they could cure the child. During the fieldworks, two women claimed that their children only *Korp* with a particular medical practitioner and that when they sought treatment from other doctors, it did not work. Similarly, a man said that his child *Korp* only with the medicines from a particular Health Centre because the child was cured there. Thus, this traditional concept of *Korp* is adding more to the understanding of the complexity of people’s choice of treatment. Medical practitioners who build a *Korp* relationship with their patients may not necessary have signed a MoU with PSK, CNM, or URC.

### Pharmaceutical and parasitic familiarity

Pharmaceutical familiarity also affects patients’ choices of malaria treatment. Throughout a year’s research, patients only mentioned the names quinine and chloroquine in conversations about malaria treatment. Most villagers do not know that such anti-malarial medicines are ineffective for *P. falciparum*. Indeed, schemes like AMFm can help to increase the availability of effective anti-malarial drugs, but ACT might not be in the patients’ choice of treatment because of its unpopularity. As a private medical provider said, “here mostly people use chloroquine…they only want red pill chloroquine with a mosquito sign on it.” Similarly, chloroquine cocktails are still popular among the forest goers. During author’s visit to a village, the author saw a package of chloroquine cocktails on a table under a house. There a man told the author that he hardly ever sought malaria treatment from VMWs, health post, health centre or any private doctors. When he got a shivering fever after coming back from the forest, he would take a few intakes of chloroquine cocktails. This choloroquine cocktail can be found at grocery stores and pharmacies in Kompot, Mondulkiri, and Koh Kong city. As a grocery store owner said, “I sell the malaria cocktails; I buy them from a pharmacy in Kompot market. Forest goers often buy several intakes before going to the forest.”

With the persistent nature of anti-malaria drug resistance, malaria is perceived as a major threat to global public health, if *P. falciparum* parasite resists to the artemisinin substance, millions of lives in developing regions will be in danger. However, that fear is not shared with people who have been living in malaria endemic areas. Since malaria has been presenting in human history for decades, many malaria patients are not scared of it. They took anti-malarial medicines without any diagnostic tests. For example, a malaria patient said, ‘I bought a pack of anti-malarial medicines without any diagnostic tests. I was not scared of malaria anymore as I got it very often, so I ate anti-malarial medicines like candies.”

## Discussion

Malaria eradication programme such as AMFm and WHO containment projects are mostly based on quick technical vertical solutions. As a result, they designed a malaria eradication mechanism that is pharmaceutically centered. As the data above show, such schemes ignore health care infrastructure, pharmaceutical regulations and political willingness of the country. In this section, author is going to discuss the pharmaceutical centered to public health, an inadequate public health infrastructure, and medical ethical responsibilities. This paper is not the first to make such arguments. For instance, Biehl [13] criticizes the idea that pharmaceuticals are a core solution to public health issues given that such a model does not address the root causes of the problems: poor public health infrastructure and socio-economic inequality. Similarly, Packard [22] claims that attention must be paid to local social and economic forces that were shaping the local malaria epidemiology. This paper extends these claims through a site-specific discussion of how culture and social norms shape anti-malaria efforts.

The pharmaceutical-centered approach to public health can be explained through an aggressive expansion of pharmaceutical companies, which suggests a pill is a solution to any public health issues. At the same time, it also reflects a neoliberal understanding of problem–solution because the programme detaches from any political entanglements, and gives the market an important role in fighting malaria. With an aggressive expansion of pharmaceutical companies, many social problems are being medicalized with a ‘pill’ being seen as a solution. For example, antidepressant pills were advertised as a promise to solve the marginalization of depressed middle-class men and women in India [23]. In the malaria case management, AMFm has been implemented in Cambodia hoping that it will help to eliminate all forms of malaria. AMFm reflects on what Biehl [13] called a pharmaceutically-centered model of public health or a ‘magic bullet approach’ to health care. Such an approach centers the power of the pharmaceutical industries in the domain of public health. It is aligned with ‘humanitarian biomedicine’, because “it is funding that seeks to avoid political entanglement and tends to emphasize technical interventions over systemic changes, in order to achieve its immediate goal, of saving lives” [24].

“Despite years of post-Alma Atta rhetoric about the need for horizontal programmes, infrastructural developments, primary health care, and popular participation” [22], a quick technical vertical and pharmaceutically centered mechanism to combat malaria is still preferable than the horizontal one. AMFm is one of its kind, 90% was funded by the external body, which pours ACT into the Cambodian pharmaceutical market and lets the market work out the price to drive out all ineffective anti-malarial medicines. Such an approach neglects the existing local power structure. As Cooke and Kothari [25], asserts that if the local power structure is not tackled, a new form of tyranny will emerge and benefit will be diverted. Medical providers, who are in more powerful positions than patients, divert the benefits of the scheme away from the poor people. Thus, poor people do not benefit from the scheme as predicted. As the findings have shown, ACT is still more expensive than a chloroquine cocktail intake due to the price’s absorption in both private and public sectors.

Technocrats supporting market-based solutions often assume that the market is perfect, and people are driven by their free and rational choices. Thus, if an anti-malarial medicine with a lower cost and higher quality is made available, people will opt for it. In reality, people’s choices of treatment are dictated by socio-economic factors, local medical practices, available health infrastructure, their conceptions of the body in relations to diseases, and their understanding of disease. All of these factors help to explain why ACT is not preferable to a chloroquine cocktail for many malaria patients.

AMFm does increase the availability of ACT in Cambodia, but it is difficult to track down whether ACT is being consumed by malaria patients after a positive test because of mal-health care infrastructure, weak regulation, and unethical responsibilities of medicines providers. For example, ACT was sold without RDT or any tests in registered and unregistered outlets. Such malpractice supports Oxfam’s claim that the global subsidy of ACT through the AMFm scheme may be contrary to its intention, encouraging drug sellers to sell the medicines without proper diagnosis [26]. Moreover, pharmaceutical regulation system in Cambodia is inadequate to make sure that ACT is consumed after a RDT test, or to ensure that ACT will not flow into unregulated sector. A study of regulation frameworks in 25 low-income countries in Africa and Asia including Cambodia, has shown that pharmaceutical legislation and regulation is often inadequate and largely un-enforceable [27].

The flow of ACT into unregulated and unregistered outlets reflects the weak drug regulation, and an unwillingness to work with an unregistered sector. Although findings reveal unregistered outlets are still rampant, a high ranking health officer denied it existence. This denial makes it even more difficult for any public or private programmes to integrate the unregistered outlets and village doctors into the programmes because if they do otherwise it contradicts the official claim. As one of the public health officers said: “working with unregistered outlets or unlicensed doctors would undermine our efforts to eradicate them.” In reality, unlicensed medicine shops in Cambodia are rampant and cannot be neglected. By neglecting the informal or unregistered sector, Cambodia is neglecting one of the most important problems that should be urgently dealt with. In December 2014, there was an AIDS epidemic in a district of Battambang [28]. This epidemic started with an unlicensed doctor who used the same syringe for multiple medical injections [ibid]. Tackling malaria eradication requires resolve and the political will of the relevant authorities. As the unregistered outlets are already un-regulated and un-monitored (in many ways), without any integration programs of the unlicensed sector, the consequences for Cambodia’s public health could be dire.

The public–private practices of medical providers implicate an inadequate public health infrastructure such as low salary, lack of opportunities, accountability, and transparency. The health officers are not held accountable by people when they do not perform duties fully. The mal-practices are normalized through time on the fact that the salary is too low to live off. This blurs the medical ethical responsibilities to save lives.

## Conclusions

This paper seeks to examine the social and cultural complexities that shape anti-malarial medicines usage and practices. The data shows the sale of ACT without RDTs and the flow of ACT from regulated sectors to unregulated sector, the price absortion by medical providers in both sectors which make ACT still more expsensive than other ineffective anti-malarial treatment for *P. falciparum* like chloroquine. These findings imply that at the current state, Cambodia does not have a strong regulation mechanism or networks to enforce a national malaria policy, especially how anti-malarial drugs are being prescribed and consumed. Furthermore, the unregulated sector remains untouched because both private and public sectors refuse to integrate unregistered and unlicensed outlets or village doctors into the malaria control programme. Thus, malaria treatment is carried out without any monitoring procedure. The refusal to integrate the unregulated sector could worsen the anti-malarial drug resistance situation in Cambodia.

Mechanisms like AMFm do not only neglect the incapability of public and private institutions to control the flows of ACT, they also ignore social-cultural factors that determine patients’ choices of treatment. Those factors are pharmaceutical familiarity, body experienced sickness, patients’ social relation with healers and power relations between patients and health care providers. Such choice of treatment determines what kind of anti-malarial drugs are being consumed, and how they are being consumed. Those factors must be taken into consideration while trying to initiate the malaria controls programmes, to ensure that the most effective anti-malarial drugs are consumed after a confirmed positive test.

This article also demonstrated that malaria patients are reluctant to seek treatment from the public sector (health care centre, health post and VMWs) because the service is irregular and the public health workers cannot be held accountable for their malpractice. This reflects poor public health infrastructure in Cambodia. A historical analysis of malaria control programmes revealed that countries that do not have enough health professionals and infrastructure are more vulnerable to the resurgence of malaria [29]. Malaria is an opportunist disease waiting to regain its endemic power where it has the opportunity to do so. Thus, Nájera [ibid] recommends that to fight malaria, policymakers need a long-term commitment with a flexible strategy that includes community involvement, integration with health systems, and the development of agile surveillance systems. Malaria needs close attention, so malaria surveillance and follow up system in Cambodia must be integrated at the communal level.

There is no short-cut to combat malaria, so Cambodia needs the following tools if they want the malaria eradication to be successful. First, a well-equipped health infrastructure that includes physical infrastructures, well-trained health professionals with a strong sense of ethical responsibility, and an accountable health care system. Second, a general educational programme on drug resistance must be launched at all levels to involve different stakeholders in the battle for malaria control and eradication. Finally, unlicensed outlets and medical providers must be integrated into the programme because of their close relations and connections with local people. Some are not only seen as healers but also as counselors. Moreover, the local doctors can influence the malaria patients and their family member’s choices of treatment. The ethnical medical responsibilities must be included into the national curriculum. All of these programmes would require sufficient financial resources and long-term commitment by the Cambodian government.

## Abbreviations

ACT: artemisinin-based combination therapy
AMF: maffordable medicines facility-malaria
CNM: National Centre for Parasitology, Entomology and Malaria Control
DDF: Department of Drug and Food
DHA-PPQ: diydroarteminin and piperaquince
GFATM: Global Fund to Fight AIDS Tuberculosis and Malaria
GHI: Global health initiatives
MDR-TB: multidrug resistance tuberculosis
MoU: memorandum of understanding
PPM: Public Private Mixed Programme
PSK: Population Service Khmer
RDTs: Rapid Diagnostic tests
URC: University Research Cooperation
VMWs: Village Malaria Workers
WHO: World Health Organization
UNITAID: Joint United Nations Programme on HIV/AIDS
UNOPs: United National Office for Project
